# Altered states of leadership: mindfulness meditation, psychedelic use, and leadership development

**DOI:** 10.3389/fpsyg.2023.1151626

**Published:** 2023-07-05

**Authors:** Otto Simonsson, Cecilia U. D. Stenfors, Simon B. Goldberg, Peter S. Hendricks, Walter Osika

**Affiliations:** ^1^Department of Neurobiology, Care Sciences and Society, Karolinska Institute, Stockholm, Sweden; ^2^Department of Sociology, University of Oxford, Oxford, United Kingdom; ^3^Department of Psychology, Stockholm University, Stockholm, Sweden; ^4^Department of Counseling Psychology, University of Wisconsin–Madison, Madison, WI, United States; ^5^Department of Health Behavior, School of Public Health, University of Alabama at Birmingham, Birmingham, AL, United States

**Keywords:** mindfulness, meditation, psychedelics, psilocybin, leadership, leaders

## Abstract

**Background:**

Previous research suggests that mindfulness meditation and psychedelic substances show promise as mental health interventions, but relatively little remains known about their potential impact on leadership outcomes.

**Aims:**

This study aimed to investigate if and how mindfulness meditation and psychedelic use may impact leadership among respondents with a management position as their primary role at work.

**Methods:**

Using samples representative of the US and UK adult populations with regard to sex, age, and ethnicity, this study used quantitative and qualitative methods to examine if and how mindfulness meditation and psychedelic use may impact leadership.

**Results:**

Among respondents with a management position as their primary role at work (*n* = 3,150), 1,373 reported having tried mindfulness meditation and 559 reported having tried psychedelics. In covariate-adjusted regression analyses, both lifetime number of hours of mindfulness meditation practice and greater psychological insight during respondents’ most intense psychedelic experience were associated with describing a positive impact on leadership (ORs = 2.33, 3.49; *p*s < 0.001), while qualitative analyses revealed nuances in the type of impacts mindfulness meditation and psychedelic use had on leadership. There were several subthemes (e.g., focus, creativity, patience, empathy, compassion) that were frequently reported with both mindfulness meditation and psychedelic use. There were also unique subthemes that were more commonly reported with mindfulness meditation (e.g., improved sleep, stress reduction, calming effects) and psychedelic use (e.g., greater self-understanding, less hierarchical attitudes toward colleagues, positive changes in interpersonal attitudes and behaviors), respectively.

**Conclusion:**

Although causality cannot be inferred due to the research design, the findings in this study suggest potential complementary effects of mindfulness meditation and psychedelic use on leadership, which could inspire new approaches in leadership development.

## Introduction

There have recently been anecdotal reports of individuals using altered states of consciousness for peak performance and leadership development (Kotler and Wheal, 2017). While there may be several ways to induce altered states of consciousness, two such interventions—mindfulness meditation and psychedelics such as lysergic acid diethylamide (LSD)—have become increasingly common in society (Simonsson et al., 2020; Livne et al., 2022).Yet there is relatively little empirical data on how these interventions, especially psychedelics, might affect leadership development.

The cultivation of mindfulness through meditation or other practices has been found to influence several outcomes related to leadership (Donald et al., 2021). For instance, Urrila (2021) conducted a systematic review on mindful leadership and identified 28 leadership-related outcomes (e.g., stress reduction, sleep, creativity, emotion regulation) that may be impacted by mindfulness-based interventions, which highlights the wide-ranging potential of mindfulness training for leadership development. The studies to date on mindful leadership have, however, had relatively small sample sizes and have not utilized nationally representative samples free of significant self-selection bias. There has also been a lack of research on potential negative impacts of mindfulness training on leadership (see Britton et al., 2021 for meditation-related adverse effects; see also Purser, 2018). It is therefore important to build on previous findings by addressing these limitations.

While psychedelics are largely unexplored in the leadership literature, recent quantitative findings suggest that moderate-to-high doses of psychedelics—if administered in safe and supportive contexts—may also impact outcomes related to leadership (see also Schlag et al., 2022 for potential risks), including psychological health (Galvão-Coelho et al., 2021), creative thinking (Prochazkova et al., 2018; Wießner et al., 2022), and interpersonal attitudes and behaviors (Griffiths et al., 2006, 2008; Roseman et al., 2021). Other qualitative research indicates that regular use of psychedelics in low doses for prolonged periods (i.e., “microdosing”) may have an impact on productivity and other leadership-related outcomes (Webb et al., 2019; see also Johnstad, 2018; Fadiman and Korb, 2019; Hutten et al., 2019). It is thus possible that psychedelics could be used as a potential tool for leadership development, but little remains known about the direct link between psychedelic use and leadership outcomes.

Previous research indicates that there may be parallels between the neurophysiology and phenomenology of altered states induced by mindfulness meditation and psychedelic use (Millière et al., 2018). Other research suggests that mindfulness meditation and psychedelic use may exert complementary long-term effects and could, in theory, be used in conjunction to amplify and prolong beneficial outcomes (Heuschkel and Kuypers, 2020). No study has so far investigated, however, in what ways the potential effects of mindfulness meditation and psychedelic use on leadership development might overlap and contrast with each other.

To address the gaps in the literature, the present study aimed to investigate the perceived impact of mindfulness meditation and psychedelic experiences on leadership at work. Using samples representative of the US and UK adult populations with regard to sex, age, and ethnicity (*N* = 9,732), we used quantitative and qualitative analyses to examine if and how mindfulness meditation and psychedelic use may have influenced leadership among respondents with a management position as their primary role at work. The perceived impact of mindfulness meditation and psychedelic use, respectively, was also compared to better understand in what ways such self-reported effects might overlap and contrast with each other.

## Materials and methods

The respondents were recruited in August 2022, through Prolific Academic,^1^ which is a platform that facilitates study participant recruitment for researchers. The platform offers representative samples of two national populations—the United States and the United Kingdom—that are stratified on three census-matched factors: sex (Male, Female), age (18–27, 28–37, 38–47, 48–57, 58+), and ethnicity (White, Mixed, Asian, Black, Other). Previous research suggests that Prolific Academic provides high data quality, relative to other recruitment platforms (Peer et al., 2017, 2021). In this study, we used Prolific Academic’s representativeness function to recruit US (*N* = 4,867) and UK (*N* = 4,865) residents who were 18 years or older. The study description did not mention psychedelics to avoid potential self-selection bias. The respondents were asked questions about demographic characteristics, employment status, mindfulness meditation, and psychedelic use. This study was part of a larger survey and respondents were paid £0.9 for completing the full survey. Study procedures were determined to be exempt from review by the Institutional Review Board at the University of Wisconsin–Madison.

### Measures

#### Demographic characteristics

All respondents were asked to report their age in years, gender [male, female, transgender (male to female), transgender (female to male), non-binary gender, other], educational attainment (Bachelor’s degree or higher, no Bachelor’s degree), degree of religiosity (1—“Not at all religious” to 5—“Very religious”), and political affiliation (Democratic Party or Republican Party for US respondents, Remain or Leave for UK respondents).

#### Management position

All respondents were asked whether they were currently employed or not. The respondents who reported current employment were asked to select the response option that best described their primary role at work:

- Board Member
- C-Level (e.g., CEO or Managing Director, CFO, COO)
- Senior Management
- Other Management
- None of the above

#### Mindfulness meditation

The respondents were asked whether they had ever tried mindfulness meditation, including Vipassana, Zen Buddhist meditation, Mindfulness-Based Stress Reduction, and Mindfulness-Based Cognitive Therapy. Those who reported that they had ever tried mindfulness meditation were asked to estimate their total lifetime number of hours of mindfulness meditation practice (0–10, 11–100, 101–500, 501–1,000, 1,001–5,000, 5,001+). If respondents reported that they had ever tried mindfulness meditation and they had a management position as their primary role at work (Board Member, C-Level, Senior Management, Other Management), they were also asked to write a few sentences describing if and how mindfulness meditation had impacted their leadership at work.

#### Psychedelic use

The respondents were asked whether they had ever used drugs, including any of the following psychedelics: N,N-Dimethyltryptamine (DMT), ayahuasca, psilocybin, LSD, mescaline, peyote, or San Pedro. Those who reported that they had ever used psychedelics were asked to think back on their most intense experience using a psychedelic and complete the Psychological Insight Questionnaire (Davis et al., 2021; Cronbach’s alpha = 0.97 in the sample of respondents who reported having a management position and having tried psychedelics), which has been designed to capture psychologically insightful experiences that might occur during the acute psychedelic experience. If respondents reported that they had ever used psychedelics and they had a management position as their primary role at work (Board Member, C-Level, Senior Management, Other Management), they were also asked to write a few sentences describing if and how psychedelics had impacted their leadership at work.

### Data analyses

The responses were coded independently by two authors (OS and WO). First, each participant’s text description was coded primarily as (1) no impact on leadership, (2) positive impact on leadership, or (3) negative impact on leadership. If the text description was perceived as contradictory or did not clearly indicate a positive or negative impact on leadership, it was coded as no impact on leadership. Once the independent analyses were finished, inter-assessor verification was carried out to ensure that consensus was reached on the coded categories. Logistic regression was used to examine the association between the respondents’ lifetime number of hours of mindfulness meditation practice and the perceived impact of mindfulness meditation on leadership (0 = no impact on leadership, 1 = positive impact on leadership). Logistic regression was also used to examine the association between psychological insight during respondents’ most intense experience using a psychedelic and the perceived impact of psychedelics on leadership (0 = no impact on leadership, 1 = positive impact on leadership). The independent variables were z-scored to standardize values and make comparison easier. Negative impact on leadership was not evaluated in regression models due to low counts in both the mindfulness meditation (*n* = 3) and psychedelic use (*n* = 8) categories. However, sensitivity analyses were conducted with no impact on leadership and negative impact on leadership combined into one category. The logistic regressions included a number of control variables that broadly correspond with those used in a previous investigation (Forstmann et al., 2020): age (recoded as: 18–27, 28–37, 38–47, 48–57, 58+), gender (recoded as: male, female, other), educational attainment (no Bachelor’s degree, Bachelor’s degree or higher), degree of religiosity (not at all religious, a little religious, moderately religious, quite religious, very religious), and political affiliation (Democratic Party, Republican Party, Remain side, Leave side). Lifetime use of other drugs (i.e., alcohol, nicotine products, cannabis products, MDMA, major stimulants, illicit narcotic analgesics/opioids, illicit benzodiazepines and barbiturates, inhalants, and other substances), country of residence (US, UK), and management position (Board Member, C-Level, Senior Management, Other Management) were included as control variables in sensitivity analyses. Second, reflexive thematic analysis was used to identify and analyze patterns in the qualitative data set. The analytical process followed six steps (Braun and Clarke, 2006, 2012): (1) familiarizing oneself with the data by reading and re-reading the text descriptions; (2) producing initial codes through coding of each respondents’ response; (3) organizing the codes into broader themes that were relevant to the research question; (4) reviewing the broader themes by adjusting and developing the initial themes identified in step three and re-reading the data associated with each theme and considering whether the data supported it; (5) defining and naming themes by identifying key features of each theme; and (6) writing up the results. Once the independent analyses were finished, inter-assessor verification was carried out to ensure that consensus was reached on the identified thematic categories.

## Results

Table 1 shows descriptive statistics for respondents in a management position (*n* = 3,150; 32.4% of total study sample). As shown in the table, 1,373 of respondents in a management position reported having tried mindfulness meditation (14.1% of total study sample; 43.6% of management sample), 559 reported having tried psychedelics (5.7% of total study sample; 17.7% of management sample), and 334 reported having tried both (3.4% of total study sample; 10.6% of management sample). A total of 396 responses on mindfulness meditation and leadership were coded as no impact (28.8%), 974 were coded as positive impact (70.9%), and 3 were coded as negative impact (0.2%). A total of 324 responses on psychedelics and leadership were coded as no impact (58.0%), 227 were coded as positive impact (40.6%), and 8 were coded as negative impact (1.4%).

**Table 1:** Descriptive statistics of respondents in a management position.

	All (*n* = 3,150)	Mindfulness meditation (*n* = 1,373)	Psychedelic use (*n* = 559)	Both (*n* = 334)
Age	*M* = 42.1 *SD* = 12.5	*M* = 41.6 *SD* = 12.4	*M* = 41.5 *SD* = 11.6	*M* = 40.9 *SD* = 11.4
Male	1,836 (58.3%)	691 (50.3%)	368 (65.8%)	200 (59.9%)
Bachelor’s degree or higher	2,223 (70.6%)	1,029 (75.0%)	367 (65.7%)	227 (68.0%)
Not at all religious	1,704 (54.0%)	745 (54.3%)	375 (67.1%)	224 (67.0%)
Political affiliation				
Democrats	957 (30.4%)	488 (35.5%)	278 (49.7%)	188 (56.3%)
Republicans	506 (16.1%)	198 (14.4%)	85 (15.2%)	42 (12.6%)
Remainers	1,218 (38.7%)	521 (38.0%)	143 (25.6%)	84 (25.1%)
Leavers	469 (14.9%)	166 (12.1%)	53 (9.5%)	20 (6.0%)
US residence	1,463 (46.4%)	686 (50.0%)	363 (64.9%)	230 (68.9%)
Primary role at work				
Board member	100 (3.2%)	45 (3.3%)	14 (2.5%)	7 (2.1%)
C-level	295 (9.4%)	155 (11.3%)	72 (12.9%)	51 (15.3%)
Senior management	690 (21.9%)	289 (21.1%)	118 (21.1%)	63 (18.9%)
Other management	2,065 (65.6%)	884 (64.4%)	355 (63.5%)	213 (63.8%)
Impact				
No impact	…	396 (28.8%)	324 (58.0%)	…
Positive impact	…	974 (70.9%)	227 (40.6%)	…
Negative impact	…	3 (0.2%)	8 (1.4%)	…

Percentages are rounded to the closest decimal point. All, all respondents in a management position. Both, respondents who reported having tried both mindfulness meditation and psychedelics.

Table 2 presents results from logistic regressions testing the associations of lifetime number of hours of mindfulness meditation practice and psychological insight during respondents’ most intense psychedelic experience with leadership impact. As demonstrated in the table, greater lifetime number of hours of mindfulness meditation practice was associated with a higher likelihood of describing a positive impact on leadership. Similarly, reporting greater psychological insight scores was associated with a higher likelihood of describing a positive impact on leadership. Sensitivity analyses revealed broadly the same results for mindfulness meditation and psychedelics, respectively.

**Table 2:** Unadjusted and adjusted logistic regression models.

	Positive impact versus no impact on leadership				
	OR (CI 95%)	*p*	aOR (CI 95%)	*p*	*n*
Lifetime mindfulness meditation practice	2.27 (1.93–2.67)	<0.001	2.33 (1.97–2.76)	<0.001	1,370
Psychedelic-induced psychological insight	3.37 (2.69–4.21)	<0.001	3.49 (2.73–4.47)	<0.001	551

OR, Odds Ratio; aOR, adjusted Odds Ratio, controlling for age, gender, educational attainment, degree of religiosity, and political affiliation. Lifetime mindfulness meditation practice and psychedelic-induced psychological insight were entered into the model separately. In sensitivity analyses, the results remained significant and in the same direction.

### Thematic analyses

Based on the analysis of the text descriptions, we identified four main themes that emerged in the texts on both mindfulness meditation and psychedelic use: (1) wellbeing and health; (2) presence and awareness; (3) productivity and performance; and (4) interpersonal attitudes and behaviors. Each theme is described below and exemplified with quotations from the respondents. Due to low counts on negative impacts, such quotation examples are included under a general theme on negative impacts.

#### Mindfulness meditation

##### Wellbeing and health

The thematic analysis revealed that wellbeing and health was a common theme across many respondents who practiced mindfulness meditation. For example, many respondents described how mindfulness meditation had a calming effect on them and had also helped them to manage stress and anxiety, which positively impacted their sleep and wellbeing. This made them more effective in their leadership and more resilient at work, especially in the midst of challenging and stressful situations.

*“When I was feeling really stressed, mindfulness [meditation] helped me to feel more relaxed. This in turn helped me to sleep better and then be a better leader.”* 34, Female, Other Management.

*“Mindfulness [meditation] helps me reduce my stress and anxiety levels generally. At work, this has helped me perform better as a leader in stressful situations. I’m able to approach difficult situations with more conscious thought.”* 25, Male, Other Management.

*“As someone who has dealt with anxiety and depression over a lifetime, mindfulness meditation has become an essential part of my self-care toolbox. I meditate in the morning to calm my mind and body rather than getting hyped up on the minutia of what tasks lie ahead in the day. As my mind is cleared, I find I am better able to process the unexpected in my day without anxiety. I meditate in the evening essentially for the same reason, to clear my mind of the day’s stressors and prepare for a restful sleep. These practices have made me a much more effective leader at work because I find I am overall less stressed and far better able to give my team individual attention and listen to them fully. We have actually incorporated meditation at our workplace and many of my team members express thankfulness for the changes it has brought to their lives both in and outside of work.”* 54, Female, Senior Management.

*“Mindfulness meditation makes me [calmer and more] focused at work. I can deal with situations in a controlled way and therefore can be the leader that others can be confident in following.”* 42, Male, Senior Management.

*“[Mindfulness meditation] has put my mind at ease, making leading others easier and less stressful.”* 20, Female, Board Member.

##### Presence and awareness

Another common theme among respondents was greater presence and awareness as a result of mindfulness meditation. For instance, respondents frequently reported more decentering, less reactivity, and greater awareness of thoughts, feelings and behaviors. This supported them in their emotion regulation and self-understanding, which, in turn, had positive downstream effects on performance and interpersonal behavior at work.

*“Mindfulness meditation has helped me become more patient and understanding in my leadership at work. I am able to separate myself from uncomfortable emotions, such as anger and irritability. I try to become more mindful and observe what is actually going on without the filter of these emotions.”* 30, Female, Other Management.

*“[Mindfulness meditation] helps me to remain observant of my thoughts and feelings in a way that promotes response rather than reaction and allows me to stay focused on important tasks.”* 43, Male, Other Management.

*“I think [mindfulness meditation] has allowed me to become more aware of the impact of my actions and words. It has influenced the way I respond to my peers and to myself.”* 44, Female, Other Management.

*“[Mindfulness meditation] helps de-stress and de-escalate my feelings and allows me to think critically about a situation instead of emotionally. Like when a subordinate makes a mistake that directly impacts me and my performance. A few minutes of mindful meditation lets me separate myself from my emotions and be able to coach my employee in a beneficial and logical way. It’s like it lets my logic and reason take over instead of emotion and feeling.”* 41, Male, Other Management.

*“[Mindfulness] meditation has provided me with a better understanding of myself and my feelings. It has allowed me to be more accepting of change and differences between people. It has also allowed me to feel more confident around others which helps in leadership, especially when making presentations or leading a team.”* 29, Female, Senior Management.

*“I use mindfulness [meditation] to enhance my effectiveness and performance including becoming more aware of my personal limits of performance. Specifically, enhanced levels of self-awareness, attention and emotion regulation were applied as mechanisms of change to improve my effectiveness.”* 47, Male, C-Level.

##### Productivity and performance

Mindfulness meditation was described by several respondents as a helpful tool for productivity and performance at work. While enhanced focus at work was widely mentioned as a result of mindfulness meditation, there were many other positive outcome descriptions, including improved priority-setting, problem-solving, creativity, and overall productivity for oneself and one’s team.

*“For me, I feel that my mindfulness [meditation has] broadened my perspectives on things, has helped me to encourage my team’s individual strengths to create better performance as a group, and has brought greater depth to my compassion.”* 64, Female, Other Management.

*“Using mindfulness meditation, I’m able to control my anxiety and stay calm at work. This has helped me keep on task and has improved my overall productivity as a leader at work. I’m able to make good job-related decisions on a day-to-day basis.”* 32, Male, Other Management.

*“Mindfulness meditation has [helped] in reducing stress and anxiety and makes me focus on things that really matter at work, which makes me effective in my leadership at work.”* 30, Female, Other Management.

*“Mindfulness meditation has helped me to concentrate and be creative as a leader at work.”* 36, Male, Senior Management.

*“I have used [mindfulness meditation] to calm down. I usually do it when I need to focus and be more productive at my work.”* 48. Male, C-Level.

##### Interpersonal attitudes and behaviors

Many respondents described how mindfulness meditation positively influenced their interpersonal attitudes and behaviors. While empathy, compassion and patience were mentioned regularly, there was also a recurring theme around a more collegial and egalitarian approach to leadership.

*“[Mindfulness meditation] helps me to be calm and not critical of others. I am able to lead with compassion.”* 69, Female, Other Management.

*“My [mindfulness meditation] practice has enabled me to not be so stressed in leadership roles and to be able to lead compassionately, without forcing authority on my subordinates.”* 59, Female, Other Management.

*“Mindfulness meditation has helped me become a gentle, more empathetic leader. I now enjoy collaborative projects, whereas before I had an individualistic approach to problem-solving. I actively listen to the needs of others within my organization, and take a more compassionate, hands-on approach to teaching, training, and leading - and I owe much of my professional growth to the daily practice of mindfulness meditation.”* 39, Female, Other Management.

*“[Mindfulness meditation has] made me more aware of the importance of treating others with compassion and equality. [It has] made me calmer in the workplace and more able to deal with problems effectively.”* 64, Male, Senior Management.

*“Mindfulness meditation has [helped] me be more empathetic and compassionate at work and develop a collegial approach to management. I do not use an autocratic management approach - that approach does not let employees develop to their full potential.”* 79, Male, C-Level.

*“I feel [mindfulness meditation] made me more connected, focused and patient. The new, more compassionated way of leading and inspiring people seems to be contagious among co-workers.”* 38, Male, Board Member.

##### Negative impact

There were a few responses that were coded as negative impact, but no discernible themes emerged due to low counts.

*I do not know if I can really say that it has impacted my leadership at work. For me, [mindfulness] meditation causes bad memories to surface and then I dissociate, which I do not find too helpful.* 49, Female, Other Management.

*It’s been completely useless. I found it more stressful to do it. I prefer physical exercise.* 37, Male, C-Level.

#### Psychedelic use

##### Wellbeing and health

The thematic analysis revealed that wellbeing and health was a recurring theme among those who reported lifetime psychedelic use. For instance, many respondents reported developing a more loving and caring relationship toward themselves and healing from depression, anxiety, or past trauma. The increase in wellbeing and health, in turn, was described as helping the respondents to lead and help others at work.

*“It made me a much calmer and happier person.”* 48, Female, Other Management.

*“My experience with psychedelics has contributed to my creativity and also helped reduce issues with depression which negatively impacted my leadership skills in the past.”* 34, Male, Other Management.

*“My experience with [psychedelics] allowed me to grow as a human and really deal with all the pain I had experienced and trauma. Even though the growth was uncomfortable, it was necessary for me to reach my full potential and be there for others in a leadership role.”* 21, Female, Other Management.

*“It has relieved stress when I [use psychedelics] occasionally, thus making me enjoy work much more.”* 33, Male, Senior Management.

*“Psychedelics have made me more confident in dealing with who I am and all my good and bad parts. It made me put my guard down and stop being such a perfectionist and realize that it’s okay to make mistakes!”* 27, Female, C-Level.

*“I have benefited from psychedelics by being able to work through past trauma and learning to love myself. That impacts my work because I feel more connected to people and the universe and I think that helps me to help my clients.”* 50, Female, C-Level.

##### Presence and awareness

There was a general theme around presence and awareness across respondents, in particular around self-understanding and psychological insight. The use of psychedelics was frequently reported to have led to more presence at work and a heightened awareness and understanding of oneself and others. This was often described as leading to more effective leadership.

*“I feel like trying [psychedelics] has impacted my leadership at work positively. I became more aware of my actions and thoughts. I was also able to understand other people more by experiencing something completely new.”* 26, Female, Other Management.

*“It gave me better insight into who I am and how I operate. It showed me there are more important things than work.”* 68, Male, Other Management.

*“I was better able to work calmly and slowly, even mindfully. I was keenly aware of myself and detached from the worries around me. I was connected on a deeper level to myself and my loved ones, pets, friends. I was a slower, more careful worker and a less stressed person overall.”* 29, Female, Senior Management.

*“It allows me to find empathy in almost anything and anyone. I should add that I probably do a heroic dose every 4 months because it resets my brain. My apathy and depression go away and I am a much more present boss. Present is the key word.”* 49, Male, Senior Management.

##### Productivity and performance

The respondents commonly reported benefits of psychedelic use related to productivity and performance. For instance, several respondents attributed a greater capacity for creativity and out-of-the-box-thinking to their use of psychedelics, but there were also a few reports of enhanced focus and problem-solving.

*“Psychedelics improved my leadership skills by allowing me to be more creative and open-minded. I believe the use of such substances may improve the overall cognitive function of the brain.”* 50, Male, Other Management.

*“Psychedelics have allowed me to be less biased and come from a place of common ground when working with subordinates and upper management alike. They have given me a sense of scale and of what really matters and of how to deal with issues in a manner appropriate to the magnitude of the issue.”* 23, Male, Other Management.

*“My experiences with psychedelics [have] generally improved my leadership at work. It has allowed me to become more hyper focused and allowed me to see things in a new light. It has allowed me to become less stressed and work better in my organization.”* 26, Male, Senior Management.

*“It made me examine myself and the world around me in more compassionate ways while recognizing personal complexities that contribute to effectiveness and productivity in a work setting. People are people, after all.”* 31, Non-Binary Gender, C-Level.

*“It strengthened my ability to see a problem from the outside looking in. Having the skill to remove one’s self from a situation is paramount to finding an objective solution.”* 48, Male, C-Level.

*“I’ve felt more open to creative ideas that I was previously unable to visualize. I’ve also been much more compassionate and empathetic since my first major trip. I feel as though these things have helped me become a better, more understanding leader.”* 44, Male, C-Level.

##### Interpersonal attitudes and behaviors

The interpersonal attitudes and behaviors theme was perhaps the most salient topic in relation to psychedelic use. Not only did respondents regularly mention compassion, empathy, forgiveness, and patience in relation to their coworkers and others, but the use of psychedelics was commonly reported to have facilitated a less hierarchical attitude toward colleagues and a closer bond—and more connectedness—with their teams at work.

*“It made me realize that we are all interconnected and one person’s actions will eventually effect everyone. I try to lead with compassion and flexibility. Since we are all connected, it makes no sense to be hard with someone.”* 45, Female, Other Management.

*“It has provided a lasting perspective that I can consider, which is the truth that all people want love and validation. I can use this knowledge to build [up those] around me to grow as a team.”* 26, Male, Other Management.

*“It has allowed me to see the bigger picture and not be harsh on employees. I realize everybody is needed for their different skills to make the wheels go round. Everybody plays their part and everybody is equal.”* 38, Male, Other Management.

*“My experiences with psychedelics have only really affected my work as a leader in the sense that I believe they made me more compassionate, empathetic, and understanding. These qualities allow me to be a more effective leader by fostering an environment in which people can thrive and build their skills comfortably.”* 26, Male, Other Management.

*“My previous experiences [with] psychedelics have fundamentally changed my interpersonal behavior by making me much more empathetic to other people’s perspectives. This has probably made a more tolerant manager than I would otherwise be.”* 45, Male, Other Management.

*I think it opened me up to the possibility that I am not always right and that I do not have to be, so I am more open to feedback and constructive criticism. I understand that no one is above or below anyone, so I do not engage in hierarchical supervision behaviors or thoughts.* 46, Female, Other Management.

*“I feel a greater sense of unity with my team members.”* 29, Male, Senior Management.

*“It’s a while since I have done any psychedelics but I feel it gave me a greater understanding of life itself and one of those lessons is to not judge others so harshly throughout life. In the work environment it can get stressful and it’s easy to cast judgment but in the end we are all the same, going through our own problems that no one else knows anything about and sometimes this can impact the things we do or say. It’s difficult at times to have this understanding [or] take this approach in a managerial position but I believe it’s helped form a closer work relationship with my colleagues which in return has boosted performance in the business.”* 36, Male, C-Level.

##### Negative impact

There were a few responses that were coded as negative impact, but no discernible themes emerged due to low counts.

*It made my work harder and I wasn’t feeling good.* 26, Male, Other Management.

*It has taken me quite a while to recover from the after effects, so it becomes difficult to focus on work.* 26, Male, Other Management.

## Discussion

Using representative samples of the US and UK adult populations with regard to sex, gender and ethnicity, this study applied quantitative and qualitative analyses to examine if and how mindfulness meditation and psychedelic use might impact leadership among respondents with a management position as their primary role at work. The quantitative analyses revealed that lifetime number of hours of mindfulness meditation practice was associated with a higher likelihood of describing a positive impact on leadership and greater psychological insight during respondents’ most intense psychedelic experience was associated with a higher likelihood of describing a positive impact on leadership. In the qualitative analyses, no discernible themes emerged from the responses coded as negative impact due to low counts, but the analyses assessing positive impact on leadership revealed four overarching themes that were common to both mindfulness meditation and psychedelic use: (1) wellbeing and health; (2) presence and awareness; (3) productivity and performance; and (4) interpersonal attitudes and behaviors. There were notable overlaps and differences within these themes when comparing mindfulness meditation and psychedelic use, which suggests that these two interventions may produce comparable and also complementary effects on leadership at work. While there were several subthemes (e.g., focus, creativity, patience, empathy, compassion) that were frequently reported with both mindfulness meditation and psychedelic use, there were a few that were relatively unique to mindfulness meditation and psychedelic use, respectively. For example, improved sleep was commonly reported among respondents who had engaged with mindfulness meditation, but sleep was not mentioned in any response among those who had used psychedelics. Another notable difference was the extent to which respondents who had engaged with mindfulness meditation reported stress reduction and calming effects as compared to those who had used psychedelics. It was, however, more common for respondents who had used psychedelics to report greater self-understanding and also positive changes in interpersonal attitudes and behaviors than those who had used mindfulness meditation. These identified overlaps and differences contribute to the emerging research literature on the potential complementary effects of mindfulness meditation and psychedelic use (Millière et al., 2018; Heuschkel and Kuypers, 2020; Payne et al., 2021; see also Simonsson and Goldberg, 2022; Simonsson et al., 2023).

There was a higher prevalence of responses coded as positive impact among respondents who reported mindfulness meditation practice (70.9% versus 40.6% of those who reported psychedelic use) and a higher (though still low) prevalence of responses coded as negative impact among respondents who reported lifetime psychedelic use (1.4% versus 0.2% of those who reported mindfulness meditation use). Such differences do not necessarily reflect a higher risk–benefit ratio for mindfulness meditation relative to psychedelic use in leadership development. It is possible, for example, that those who reported psychedelic use had taken these substances in unsupportive contexts, in low doses, or in combination with other substances, all of which could have influenced responses. The same could also be true for those who reported mindfulness meditation use.

These findings must be considered in light of several limitations with the research design. First, although the responses were coded by two researchers independently, it is possible that the respondents themselves or other researchers would disagree with the coding of the responses. It may also have been useful to have utilized more than two researchers coding the responses to reduce potential bias. Second, the respondents were asked a single qualitative question about leadership and were not asked to provide additional details about their use of mindfulness meditation or psychedelics that could have been important to evaluate (e.g., age of first use, context of use, delivery of mindfulness training, dose of psychedelics). No questions were asked, for example, about whether the use of mindfulness meditation or psychedelics preceded work experience in a management position, which would have been important to better understand temporal sequence. The single qualitative question also did not ask about the durability of the potential effects (e.g., transient, lasting). Third, respondents who reported current employment were asked to select the response option that best described their primary role at work, but no additional questions were asked that could have been useful (e.g., company size, staff responsibilities). Fourth, the findings cannot be used to infer causality, due to the cross-sectional design of the study. Fifth, the responses were collected from a single survey and were both retrospective and self-reported, which increases the likelihood of response biases (Podsakoff et al., 2003). It is possible, for instance, social desirability influenced responses (Van de Mortel, 2008), especially those related to substance use (Latkin et al., 2017). Sixth, the samples were stratified to reflect the US and UK populations with regards to sex, age and ethnicity, but it is possible that individuals with negative experiences of mindfulness meditation or psychedelic use are less represented on online recruitment platforms such as Prolific Academic. If this were indeed true, it would limit the generalizability of the findings. Future research should utilize in-depth qualitative interviews and longitudinal research designs, including randomized controlled trials, to better understand if, for whom, and under what circumstances mindfulness meditation and psychedelic use might influence leadership development. It would be particularly important to investigate potentially harmful effects of mindfulness meditation and psychedelic use, especially in relation to the workplace.

## Conclusion

While the findings in this study should be considered preliminary due to the limitations of the research design, the results suggest that mindfulness meditation and psychedelic use may produce comparable and also complementary effects on leadership at work. If replicated in future studies with more rigorous research designs (e.g., randomized controlled trials), such findings could lead to the development of novel training programs that combine both mindfulness meditation and psychedelics to improve leadership at work.

## Data availability statement

Data and code are available at figshare: https://doi.org/10.6084/m9.figshare.23541459.v1.

## Ethics statement

Study procedures were determined to be exempt from review by the Institutional Review Board at the University of Wisconsin–Madison. The patients/participants provided their written informed consent to participate in this study.

## Author contributions

OS conceptualized and designed the study. OS and WO analyzed the data. OS wrote the manuscript, with comments from CS, SG, PH, and WO. All authors contributed to the article and approved the submitted version.

## Funding

OS was supported by Ekhaga Foundation and Olle Engkvist Foundation. SG was supported by a grant (K23AT010879) from the National Center for Complementary and Integrative Health. Support for this research was also provided by the University of Alabama at Birmingham School of Public Health, Swedish Research Council for Sustainable Development (FORMAS; FR-2018-0006; FR-2018-00246), Forte (2020–00977), and the University of Wisconsin–Madison Office of the Vice Chancellor for Research and Graduate Education with funding from the Wisconsin Alumni Research Foundation and with funding from the Wisconsin Center for Education Research.

## Conflict of interest

PH was on the scientific advisory board of Bright Minds Biosciences Ltd., Eleusis Benefit Corporation, and Reset Pharmaceuticals Inc. OS was a co-founder of Eudelics AB.

The remaining authors declare that the research was conducted in the absence of any commercial or financial relationships that could be construed as a potential conflict of interest.

## Publisher’s note

All claims expressed in this article are solely those of the authors and do not necessarily represent those of their affiliated organizations, or those of the publisher, the editors and the reviewers. Any product that may be evaluated in this article, or claim that may be made by its manufacturer, is not guaranteed or endorsed by the publisher.

## Footnotes

^1^ https://app.prolific.co
